# Images of Cecal Volvulus From a Strangulating Fallopian Tube: A Case Report

**DOI:** 10.4021/jocmr661w

**Published:** 2011-09-26

**Authors:** May C. Tee, Tracy Chandler, Julia Hlynsky, Abdul Aleem

**Affiliations:** a Department of Surgery, University of British Columbia, Vancouver, Canada; b Department of Diagnostic Radiology, University of British Columbia, Vancouver, Canada; c Department of Family Medicine, University of British Columbia, Vancouver, Canada

## Abstract

An unusual case of cecal volvulus arising from a strangulating fallopian tube is presented. The etiology, diagnosis, and management guidelines of this infrequent cause of large bowel obstruction are reviewed. Computed tomography images are included, which demonstrate key features that are pathognomonic for this condition. To our knowledge, this is the first report of gynecologic adnexa giving rise to cecal volvulus.

**Keywords:**

Cecal volvulus; Gynecologic and general surgery; Intestinal obstruction

## Introduction

Cecal volvulus represents an unusual cause of large bowel obstruction and requires a mobile cecum in addition to a point of fixation for which the bowel may tort upon itself. We present a case of cecal volvulus after a presumed episode of gastroenteritis with the point of fixation being a remnant fallopian tube twisted around the base of the cecal mesentery from a remote abdominal hysterectomy. Although vomiting and prior abdominal surgery might have contributed to this intestinal torsion and obstruction, operative intervention revealed a strangulating fallopian tube as the point of fixation. Based on a thorough literature review, we believe this is the first reported case of a cecal volvulus caused by remnant adnexa from previous gynecological surgery.

## Case Report

A 62-year-old female, previously healthy with a remote history of prior lumbar spine fixation and abdominal hysterectomy, was referred for evaluation and management of ileus. She reported ingesting spoiled vegetables three days earlier resulting in nausea, vomiting, and non-bloody diarrhea. She was seen by her family physician and prescribed ciprofloxacin, dimenhydrinate, and loperamide for presumed gastroenteritis. She was not on any other regular medications. Since that time, her nausea and vomiting had resolved; however, she failed to pass any flatus or stool and complained of increasing abdominal pain and distention. Abdominal exam revealed a distended abdomen with prior Pfannensteil incision, minimal bowel sounds, tympanic percussion, but no evidence of peritonitis. She was mildly febrile although hemodynamically stable. Laboratory work revealed leukocytosis with left shift and abnormal renal function. Plain abdominal films demonstrated diffusely distended loops of bowel with some air fluid levels.

**Figure 1:**
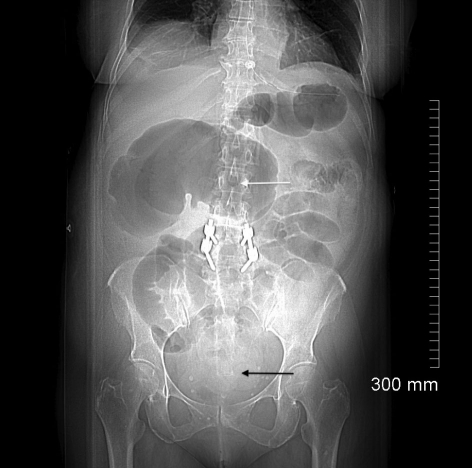
CT scout image demonstrates a dilated cecum in the right upper quadrant producing the classic ”coffee bean“ sign (white arrow). There are multiple dilated loops of small bowel present centrally within the abdomen with a paucity of gas in the distal colon (black arrow) suggestive of a complete intestinal obstruction. This patient had spinal instrumentation consistent with her previous history of spine surgery.

**Figure 2:**
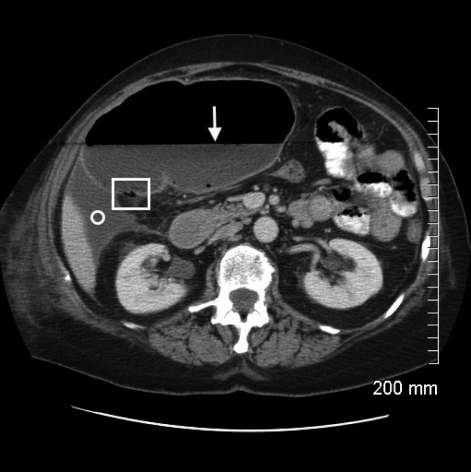
Axial CT image with intravenous and oral contrast through the level of the mid-abdomen demonstrates massive dilatation of the cecum. There is a prominent air-fluid level (white arrow) and multiple locules of air are noted in the dependent portion of the wall consistent with pneumatosis intestinalis (square). However, no extraluminal free air is present to suggest frank perforation and the wall of the cecum continues to enhance uniformly. The associated perihepatic free fluid (circle) is consistent with a high-grade type of intestinal obstruction.

**Figure 3:**
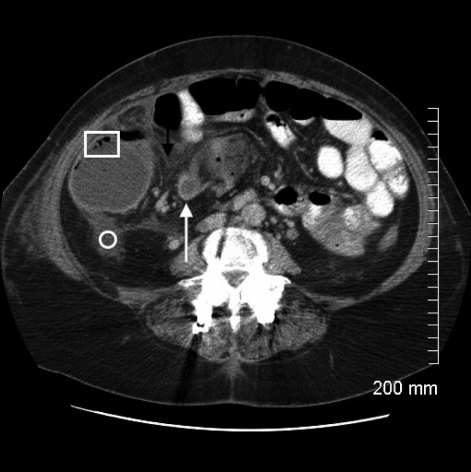
Axial CT image demonstrates twisting of the mesenteric vessels producing the ”CT whirl“ sign, which is highly specific for intestinal volvulus (white arrow). Again demonstrated are the associated pneumatosis intestinalis (square) and pericolonic free fluid (circle).

**Figure 4:**
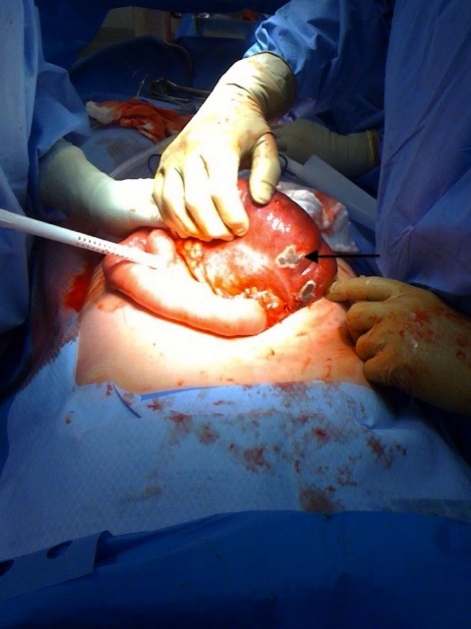
Laparotomy revealed a massively dilated cecum in the right upper quadrant consistent with radiological findings. Turbid free fluid was encountered upon entry into the abdomen. The cecum demonstrated patchy areas of ischemic necrosis (black arrow) and despite carefully twisting it free from its strangulated mesentery, remained unviable. A right hemicolectomy with primary ileocolic anastomosis was performed.

**Figure 5:**
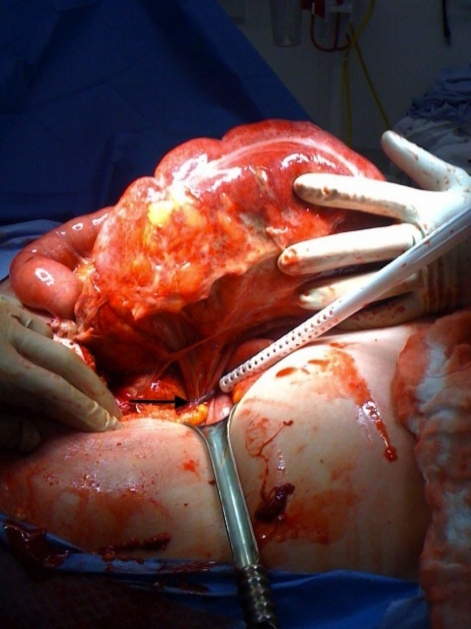
A tubular structure was found to be strangulating the cecal mesentery (black arrow). This tubular structure was gently dissected free and found to originate in the pelvis. The patient had a remote history of abdominal hysterectomy and it was unclear whether gynecological adnexae were concurrently removed. An intra-operative gynecological consult was obtained and the tubular structure was identified as the remnant right fallopian tube and confirmed on pathology. A bilateral salping-oophorectomy was performed.

**Figure 6:**
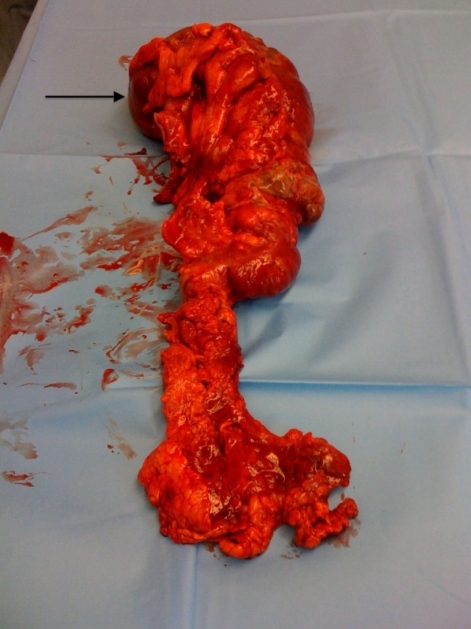
Right hemicolectomy specimen demonstrating areas of intestinal gangrene from closed loop obstruction and ischemic necrosis (black arrow indicates cecum). Pathological examination confirmed the presence of full-thickness intestinal wall necrosis. There were no intraluminal masses identified in the pathology specimen.

The patient was fluid-resuscitated until her kidney function normalized, and had nasogastric decompression for her ileus, presumed to be related to loperamide use. After 24 hours of nasogastric decompression, the patient remained obstipated. A computed tomography was ordered, which revealed closed-loop large bowel obstruction secondary to cecal volvulus associated with intestinal wall pneumatosis (Fig. 1 Fig. 2 Fig. 3). Based on the concerning lack of clinical progress and radiological findings, she was taken to the operating room urgently for laparotomy (Fig. 4 Fig. 5 Fig. 6).

Laparotomy revealed a massively distended right colon with ischemic necrosis at the cecum but no frank perforation. The etiology of this large bowel obstruction appeared to be volvulus at the level of the cecum mesentery, where the mesentery was tethered by a tubular structure identified as a right fallopian tube, and confirmed by intra-operative gynecological consultation. A right hemicolectomy with primary ileo-colonic anastomosis was performed as were bilateral adnexectomy of remnant fallopian tubes. Post-operatively, the patient was maintained on a brief course of total parenteral nutrition until her bowel function returned on the third post-operative day. She was discharged home on the sixth post-operative day without complication, tolerating a full oral diet with normal bowel function.

## Discussion

Cecal volvulus is a rare cause of intestinal obstruction, accounting for approximately 1% of all adult cases [1,2]. A volvulus occurs when bowel is twisted upon its mesenteric axis resulting in intestinal obstruction [3-6]. The site of large bowel volvulus may be localized to the cecum, terminal ileum, or ascending colon in up to 30% of these cases [1,2,6]. Cecal volvulus requires a mobile proximal colon and fixation of the bowel at a specific point, which creates a fulcrum for bowel to twist upon itself [1,4,5]. Fixation points for cecal volvulus are thought to be related to abdominal adhesions, recent surgical manipulation, or intra-abdominal masses [4,6]. Other precipitating factors for adult large bowel volvulus include abdominal surgery, straining, violent purging, vigorous activity, chronic constipation, high fiber diet, and intra-uterine pregnancy [2,4,5]. It is estimated that between 11-25% of the population have congenitally mobile proximal colons, typically from failed development of normal peritoneal attachments [1,2,4]. A normal rotation of 270 degrees typically occurs during fetal development; however, when this rotation is disrupted, there may be inadequate fixation to the posterior parietal peritoneum or over-rotation resulting in abnormally increased proximal colon mobility [1,7].

The clinical presentation of cecal volvulus depends on whether the episode is intermittent, acute, or fulminant [1]. Some patients will complain of crampy abdominal pain associated with distention and relief brought upon by passage of flatus or stool; these episodes are recurrent and typically represent the intermittent form of cecal volvulus [5]. Acute cecal volvulus, on the other hand, presents much like acute large bowel obstruction that may progress to ischemic necrosis, peritonitis, perforation, and septic shock [1,5]. The clinical signs and symptoms for cecal volvulus, being nausea, vomiting, obstipation, abdominal pain and distention, are not very specific for diagnosis [1,5,6]. Laboratory investigations are neither sensitive nor specific as well [1]. As with any intestinal obstruction, patients may or may not have altered bowel sounds, distention, or peritoneal signs, although an eccentric dilated mass that is tympanic to percussion has been described as a possible indicator of cecal volvulus [5].

Correct diagnosis of cecal volvulus is challenging to make on clinical grounds alone given the lack of specificity in clinical signs and symptoms. Instead, imaging in the form of plain radiographs and computed tomography have become invaluable in diagnosing cecal volvulus, with a reported accuracy greater than 90% [6]. On plain radiographs, cecal volvulus is typically diagnosed by a ”coffee bean“ deformity associated with cecal dilatation, air fluid levels, and the absence of distal colonic gas [1, 2, 4-6]. On CT images, cecal volvulus is also characterized by dilatation of the cecum as well as more specific signs of a ”bird beak“ (two limbs of bowel converging at the site of torsion) and ”CT whirl“ (spirals of collapsed bowel around engorged mesenteric vessels) [1,4,6].

Cecal volvulus can be categorized by its mechanism of torsion, which can subsequently be detected by imaging studies. In approximately 50% of patients, the cecum twists in an axial plane either clockwise or counter-clockwise and appears as a dilated loop of bowel on the right side of the abdomen [4]. The next most common form is referred to as a loop-type cecal volvulus where the cecum twists and inverts upon itself manifesting as a dilated loop of bowel in the left upper quadrant [4]. A less common variant of cecal volvulus, occurring up to 10% of the time, is referred to as a ”cecal bastule“ where the cecum folds on itself anteriorly with a dilated loop of bowel visualized in the mid abdomen [1,4,6].

Management of cecal volvulus follows the same principles regardless of the type of cecal volvulus. Although non-operative management in the form of a barium enema or colonoscopy has been described, it is generally not preferred given the high failure rate necessitating urgent operative intervention and the risk of perforation in an already compromised loop of bowel [1,7]. Surgical management provides more definitive management of cecal volvulus and may be divided into resection and non-resection methods. When obvious gangrene or ischemic necrosis is present, a cecectomy, ileocolic resection, or right hemicolectomy is warranted based on the extent of non-viable bowel [3,6,7]. The decision to perform primary anastomosis or end ileostomy is generally made on a case-by-case basis [6]. When the bowel is viable, it may be carefully untwisted and a cecopexy or cecostomy may be performed to prevent recurrent episodes of cecal volvulus [6,7]. The cecopexy is performed by plicating the cecum to the posterior peritoneum, which in essence restores it back to a more fixed position [6]. Alternatively, a cecostomy tube may also be placed, which immobilizes the cecum by adhering it onto the anterior abdominal wall; the added advantage of the cecostomy tube is that is provides a method of instant decompression of the distended cecum [6].

This unusual case of cecal volvulus highlights the risk factors for developing cecal volvulus. It also underscores the importance of early surgical consultation and intervention for presumptive ileus, which may otherwise be a consequence of bowel obstruction. The patient’s remote history of abdominal hysterectomy and vomiting secondary to gastroenteritis increased her risk for developing cecal volvulus. What is unique about this case is that rather than adhesive bands being the point of fixation for the patient’s cecal volvulus, it was a remnant fallopian tube that had wrapped itself around the cecal mesentery creating vascular compromise and ischemic necrosis of the ascending colon. The CT features of a ”coffee bean“ in the right upper quandrant on the CT scout are classic for cecal volvulus along with a massively dilated cecum and ”CT whirl“ sign seen on axial slices. The management of this patient was resection of the involved bowel with primary anastomosis, for which the patient recovered well and was eventually discharged home without complication.
